# Comparative Insights into Four Major Legume Sprouts Efficacies for Diabetes Management and Its Complications: Untargeted versus Targeted NMR Biochemometrics Approach

**DOI:** 10.3390/metabo13010063

**Published:** 2022-12-31

**Authors:** Mohamed A. Farag, Asmaa F. Aboul Naser, Ahmed Zayed, Mohamed G. Sharaf El-Dine

**Affiliations:** 1 Pharmacognosy Department, Faculty of Pharmacy, Cairo University, Cairo 11562, Egypt; 2 Therapeutic Chemistry Department, National Research Centre, Dokki, Giza 12622, Egypt; 3 Pharmacognosy Department, College of Pharmacy, Tanta University, Elguish Street (Medical Campus), Tanta 31527, Egypt; 4 Institute of Bioprocess Engineering, Technical University of Kaiserslautern, Gottlieb-Daimler-Str. 49, 67663 Kaiserslautern, Germany; 5 Pharmacognosy Department, Faculty of Pharmacy, Port Said University, Port Said 42515, Egypt

**Keywords:** biochemometrics, faba bean, flavonoids, hepatoprotective, hypoglycemia, legume sprouts

## Abstract

Interest in the consumption of seed sprouts is gradually increasing as functional foods in the modern Western diet owing to their several nutritional and health benefits. The present study aims to investigate four major legume sprouts derived from faba bean (*Vicia faba* L.), lentil (*Lens esculenta* L.), chickpea (*Cicer arietinum* L.), and fenugreek (*Trigonella foenum-greacum* L.) for their antidiabetic activity and mitigation of associated complications, i.e., oxidative stress, liver dysfunction, and lipid metabolism, compared with glibenclamide. Biochemical results presented herein further showed that the four sprouts exhibited significant hypoglycemic effects (*p* < 0.05), with improvement in decreasing of blood glucose levels at different degrees and with faba bean sprout most active at 348% improvement, compared to 364.3% for glibenclamide. Further biochemometric analysis based on a comparison between targeted versus untargeted partial least square (PLS) and regression analyses revealed that faba bean sprouts’ richness in flavonoids was a determinant key factor for such efficacy. In addition, correlation with previously investigated NMR fingerprinting aided in pinpointing other active agents, such as betaine and L-DOPA. Furthermore, the effect on serum liver enzymes, including alanine aminotransferase, aspartate aminotransferase, and alkaline phosphatase; oxidative stress markers; and lipid profiles showed significant improvement, especially in the case of faba bean sprout. The study revealed the potential health benefits of legume sprouts in the treatment of diabetes and its associated complications, as well as the potential role of biochemometrics in active agents’ identification in such a complex matrix to be considered for other functional foods investigation.

## 1. Introduction

Following Orchidaceae and Asteraceae, Leguminosae (Fabaceae) is the third largest plant family in Angiosperm taxa, including more than 760 genera and approximately 19,500 species grown throughout the world [1]. Legumes are recognized as the second most economical crop, following Poaceae or the rice family [2]. Particularly, legume seeds are the major edible part commonly consumed as vegetables and ingredients of food recipes [3]. They are considered nutritious foods, owing to their high contents of unsaturated fatty acids, proteins, vitamins, and minerals. Phenolic compounds (e.g., phenolic acids, flavonoids, and anthocyanins) demonstrate a wide spectrum of biological activities as antioxidant, antitumor, anticoagulant, anti-inflammatory, antimicrobial, cardioprotective, and others [3,4]. However, their potential benefits cannot be fully utilized, owing to several factors mainly related to their low protein digestibility, hard-to-cook effect, anti-nutrient constituents, and undesirable flavour [4]. Alternatively, legume sprouting has been documented to improve bioavailability and digestibility [5]. In addition, amino acids and GABA contents are increased by seed germination of various foods, including legume seeds. For instance, mung bean (*Vigna radiata*), where amino acids and γ-aminobutyric acid (GABA) contents increased by 8.7 and 27.9 times, respectively [6].

Legume sprouts derived from faba bean (*Vicia faba* L.), lentil (*Lens esculenta* L.), chickpea (*Cicer arietinum* L.), and fenugreek (*Trigonella foenum-greacum* L.) have been previously investigated regarding their phytochemical composition based on different analytical platforms, such as nuclear magnetic resonance (NMR), ultra-performance liquid chromatography-mass spectrometry (UPLC-MS), and gas chromatography-mass spectrometry (GC-MS) [7,8]. NMR results showed that trigonelline was abundant in all sprouts, indicating a possible vital role in the sprouting process. Additionally, isoflavones (e.g., genistein, cicerin, and daidzein) were unique constituents in chickpea sprouts, in addition to the high content of ω6-fatty acid, choline, and sucrose, while fenugreek, faba bean, and lentil sprouts were characterized by higher levels of 4-hydroxyisoleucine, L-DOPA, and acetic acid, respectively [8], Table S1. In addition, GC-MS post-derivatization and UPLC-MS analyses resulted in the identification of 78 and 303 metabolites, respectively. With relation to total flavonoid content (e.g., kaempferol-*O*-glycosides), faba bean sprouts showed the highest antioxidant activity, compared to the other investigated sprouts [7]. Such complex composition of legume sprouts, especially being rich in phytonutrients, warrant further biological studies for their potential health effects and identification of underlying active agents.

Diabetes mellitus (DM) is a metabolic disorder that is characterized by hyperglycemia resulting from insulin deprivation (Type I) or lack of cell response to insulin (Type II) [9]. Diabetic patients are further at increased risk of cardiovascular, cerebrovascular, and peripheral vascular complications. Additionally, hyperglycemia causes retinopathy, nephropathy, and neuropathy that may lead to blindness, renal failure, and amputations, respectively [10]. The liver is a crucial organ that regulates glucose, lipids, and protein metabolism. It plays a critical role in maintaining blood glucose balance by three regulatory processes: (1) insulin production by pancreatic cells, (2) glucose uptake activation by the liver, gut, and muscle tissues, and (3) hepatic glucose output inhibition [11]. Furthermore, hepatotoxicity is one of the foremost issues associated with DM [12].

Various studies have reported legumes’ antidiabetic action or their germinated seeds [13,14] and suggested mechanisms underlying the antidiabetic effect are mediated through delaying gastric emptying and inhibition of carbohydrate digestive enzymes. Moreover, seeds rich in trigonelline protect pancreatic *β*-cells, increase both insulin level and sensitivity [15], and stimulate an insulin signaling pathway [9]. The effect of legume sprouts against DM and associated complications have not been fully investigated, while they have been found to exhibit improved activity over dried seeds. For instance, the hydroalcoholic extract of germinated *Lens culinaris* Medik showed a significant reduction of blood glucose level and improved serum-lipid profiles in diabetic mice (*p* < 0.05) [13]. Yet, these studies could not correlate these activities with legume sprouts´ metabolome, nor identify the best sources of sprouts among legume seeds for these health effects.

Therefore, the current study extends our previous phytochemical analyses of various standardized legume sprouts to assess the antidiabetic potential and associated complications in streptozotocin (STZ)-induced diabetic rats. The study also aims to use the biochemometrics approach for the first time, correlating these activities with the unique metabolic profiles previously characterized based on NMR. Specifically, the application of targeted and untargeted partial least square (PLS) analysis should reveal novel insights into metabolites contributing to bioactivities, and further identify the most active legume sprouts for that effect based on a comparative approach.

## 2. Materials and Methods

### 2.1. Plant Material and Chemicals

A number of major legume seeds were chosen as model plants including broad or faba bean (*Vicia faba* L. cv. Giza 3), lentil (*Lens esculenta* L. cv. Sinai 1), chickpea (*Cicer arietinum* L. cv. Giza 88), and fenugreek (*Trigonella foenum-greacum* L. cv. Giza 2). Seeds were purchased from Food Legumes Research Department, Field Crops Research Institute (FCRI), Agricultural Research Center (ARC), Giza, Egypt. They were identified by Prof. Dr. Adel El Garhy; Agricultural Research Station, Itay El Barud, Beheira, Egypt.

STZ was purchased from Sigma-Aldrich^®^ (St. Louis, MO, USA). Glibenclamide (hypoglycemic reference drug) was obtained from Sanofi, Egypt. Kits used for the quantitative determinations of different parameters were purchased from Biodiagnostic Co. (Cairo, Egypt).

### 2.2. Sprouting Method

The sprouting process, in three biological replicates (n = 3), was carried out as previously described by Lv et al. [16] with few modifications. Seeds (100 g) were germinated in dark glass dishes lined with cotton after soaking in distilled water for 8 hours (h) at 28 °C. To prevent seed drying and microbial contamination during the germination process, seeds were moistened with distilled water every 3 h and washed twice daily, respectively. Afterwards, radicals were collected at the age of 3 days, lyophilized using a STELLAR^®^ Laboratory Freeze Dryer (Millrock 100 Technology, Inc., Kingston, NY, USA), and kept at −20 °C for further investigations.

### 2.3. Extraction, Metabolites Profiling, and Fingerprinting

In a ratio of 1:5, 20 g of dried lyophilized sprouts were finely powdered and macerated in methanol (100% *v/v*) until exhaustion for 3 times. After extracts evaporation under reduced pressure using a rotary evaporator, the obtained residues weighed 8.7, 8.2, 7.8, and 7.5 g for faba bean, lentil, chickpea, and fenugreek, respectively. The metabolites profiling, finger printing, and standardization of legume sprout extracts were carried out by UPLC-MS and qNMR, exactly as previously reported in [7,8,17,18].

### 2.4. Animals and Diabetes Induction

64 male Wistar strain albino rats (150–200 g) were obtained from the Animal House, National Research Centre, Dokki, Giza, Egypt. Rats were fed on a standard diet (El-Kahira Co. for Oil and Soap) and had free access to tap water. They were kept for 2 weeks to acclimatize to the environmental conditions (23–25 °C and 12 h light/dark cycle).

Anesthetic procedures and handling of animals complied with the ethical guidelines of the Medical Ethical Committee of the National Research Centre and Faculty of Pharmacy, Cairo University in Egypt, following the guidelines of the 18th WMA General Assembly, Helsinki, June 1964, updated by the 59th WMA General Assembly, Seoul, in October 2008 (Approval no.: 13,089, Date: 27 June 2013).

Animals were fasted overnight and diabetes was induced by a single dose of STZ (50 mg/kg body weight (b.wt)) dissolved in 0.01 M sodium citrate buffer, pH 4.4 immediately before use [19]. After injection, animals had free access to food and water. Two hours following STZ injection, rats were given 5% glucose solution overnight to counter hypoglycemic shock [20]. After three days of diabetic induction, rats were fasted overnight, and the blood glucose level was estimated. Rats with blood glucose level >300 mg/dL were considered diabetic.

### 2.5. Hypoglycemic Activity Evaluation

#### 2.5.1. Experimental Design

The rats were divided into eight groups of eight rats (n = 8) each, as follows. Group 1: Normal control rats were administered a single oral dose of 0.5 mL 1 M citrate buffer. Group 2: STZ-induced diabetic rats (50 mg/kg b.wt) and left untreated. Group 3: Diabetic rats were treated with chickpea sprouts extract. Group 4: Diabetic rats were treated with lentil sprouts extract. Group 5: Diabetic rats were treated with fenugreek sprouts extract. Group 6: Diabetic rats were treated with fava bean sprouts extract. Group 7: Diabetic rats were treated with trigonelline [21]. Group 8: Diabetic rats were treated with glibenclamide reference drug. The diabetic rat groups 3-6 were injected intraperitoneally (i.p.) daily with the sprouts extract (15 mg/kg b.wt), while group 7 with trigonelline (1.2 mg/kg b.wt) and group 8 at a daily oral dose of 5 mg/kg b.wt [22]. Treatment was carried out throughout a period of 10 days after DM induction by STZ.

#### 2.5.2. Sample Preparation

After 10 days of treatment, animals were fasted overnight (12–14 h), and blood samples (5 mL) were taken from each animal by puncture of the sublingual vein into sterilized tubes, and let stand for 10 min to clot. The serum was separated by centrifugation at 3000 rpm for 10 min. The separated serum was stored at −80 °C for further determinations for biochemical analysis. After blood collection, all rats of each group were sacrificed under ether anesthesia. The livers from different experimental normal and diabetic groups were removed immediately, weighed, homogenized in 0.9% sodium chloride (normal saline) (1:5 *w/v*), and centrifuged at 4000 rpm for 15 min. The supernatant was collected, aliquoted in Eppendorf tubes, and stored at −80 °C. The supernatants were used for further oxidative stress markers assessment.

#### 2.5.3. Determination of Blood Glucose Level

The serum glucose level was determined using the enzymatic colorimetric method reported by Cronin and Smith [23], where glucose was oxidized to gluconic acid by glucose oxidase producing hydrogen peroxide (H_2_O_2_). The produced H_2_O_2_, in the presence of peroxidase enzyme, coupled with phenol and 4-aminoantipyrine, resulted in a colored quinonimine that can be measured at 510 nm.

### 2.6. Effect of Different Treatments of Sprouts on Diabetes Complications

#### 2.6.1. Assessment of Liver Functions

- Determination of liver function enzymes

Serum alanine aminotransferase (ALT) and aspartate aminotransferase (AST) were estimated by the method of Reitman and Frankel [24], using a diagnostic kit (Bio Systems, Spain). AST and ALT were measured by monitoring the concentration of keto acids, i.e., oxaloacetate and pyruvate, hydrazone derivatives formed following the reaction with 2,4-dinitrophenylhydrazine at 520 nm. The concentration was calculated according to the following equation:

$$\frac{A\;\mathrm{Sample}-\;A\;\mathrm{Control}}{A\;\mathrm{Standard}-\;A\;\mathrm{Blank}}\times Conc.Standard$$

Additionally, serum alkaline phosphatase (ALP) was determined by the method of Thomas [25], using a diagnostic kit (Bio Systems, Spain). ALP catalyzes in alkaline medium *p*H (10) the phenyl phosphate group into phenol and phosphate. The liberated phenol was measured colorimetrically in the presence of 4-aminophenazone and potassium ferricyanide. The yellow color utilized of sample (A _Sample_) and standard (A _standard_) against reagent blank was measured at 510 nm, and enzyme activity was determined according to this equation:

Enzyme activity (IU/L) = A _Sample_/A _Standard_ × 75

#### 2.6.2. Assessment on Lipid Metabolism

- Determination of Cholesterol Level

Total serum cholesterol was estimated using the cholesterol oxidase method described by Meiattini et al. [26]. The color intensity recorded at 500 nm was directly proportional to the cholesterol concentration. The cholesterol level in the sample was calculated using the following formula:

A _Sample_/A _Standard_ × C _Standard_ = C _Sample_

while the catalytic concentration was determined using:

A _Sample_/A _Standard_ × 200 = mg/dL Cholesterol

- Determination of high-density lipoprotein cholesterol (HDL-C)

Serum high-density lipoprotein cholesterol (HDL-C) was estimated by the method of Burstein et al. [27], where low-density lipoprotein (LDL) in the sample is precipitated with polyvinyl sulphate. HDL cholesterol detected in the supernatant was measured spectrophotometrically using coupled reactions described previously with cholesterol determination. HDL cholesterol levels in the sample were calculated using the following formula:

C _Sample_ = A _Sample_/A _Standard_ × C _Standard_ × Sample dilution factor

and the catalytic concentration was calculated as:

A _Sample_/A _Standard_ × 52.5 = mg/dL cholesterol

- Determination of low-density lipoprotein cholesterol (LDL-C)

Serum low-density lipoprotein cholesterol (LDL-C) levels were determined following Assmann et al. [28]. LDL in samples was precipitated with polyvinyl sulfate. Its level was calculated in the supernatant from the difference between the total serum cholesterol determined previously and the cholesterol in the supernatant post centrifugation.

- Determination of serum triglycerides (TG)

Serum triglyceride (TG) was measured using the method of Fossati and Prencipe [29]. The assay is based on hydrolysis of TG by lipase-producing glycerol, which reacts with glycerol kinase and _L_-*α*-glycerol-phosphate oxidase, producing H_2_O_2_. The H_2_O_2_ level was monitored in the presence of peroxidase, with 4-aminoantipyrine and 4-chlorophenol as a chromogenic system. Finally, the absorbance was recorded at 500 nm, and the TG level in the sample was calculated using the general formula:

C _Sample_ = A _Sample_/A _Standard_ × C _Standard (200 mg/dL)_

#### 2.6.3. Effect on Oxidative Stress Markers

- Determination of superoxide dismutase activity

Superoxide dismutase (SOD) activity in liver tissue was assayed according to the method of Nishikimi et al. [30]. The enzyme activity was calculated according to the following equation:

$$\frac{\Delta A}{E.C}\;\times \;\mathrm{assay}\;\mathrm{volume}\;\times \;\frac{1}{n}=\mu \mathrm{mol}/\mathrm{mg}\;\mathrm{protein}$$

Δ*A* = The change in the absorbance of NADH, H^+^ per min

*E.C* = Extinction coefficient of NADH, H^+^ which is equivalent to 6.22 × 10^−3^ μmol cm^−1^

N = mg protein in the sample

- Determination of glutathione level

Glutathione (GSH) content in liver tissue was assayed following Moron et al. [31]. GSH was estimated using 5,5′-dithio-*bis*-2-nitrobenzoic acid (DTNB), which produces a stable yellow color that can be measured at 412 nm. The concentration of the sample that corresponds to the obtained absorbance was calculated from the pre-prepared standard curve (10–110 µg glutathione) and expressed as µg GSH/g tissue.

- Determination of malondialdehyde level

Malondialdehyde (MDA) levels were estimated in liver tissue according to the method of Buege and Aust [32]. The assay is based on MDA reaction with thiobarbituric acid (TBA) in acid medium (perchloric acid 10%), resulting in an orange-colored complex that can be measured at 532 nm. MDA concentration was calculated by the following equation:

$$\frac{A\;test}{E.C}\times \frac{1}{n}=\;\mu \mathrm{mol}/\mathrm{mg}\;\mathrm{protein}$$

*A_test_* = Absorbance of the test

*E.C* = Extinction coefficient of MDA = 1.56 × 10^5^ M^−1^ cm^−1^

*n* = mg protein in the sample

### 2.7. Determination of Total Protein in Tissue Homogenate

Total protein was assayed in tissue liver homogenate and serum according to Bradford [33]. The assay depends on the binding of Coomassie Brilliant blue dye to protein, producing a blue complex measured colorimetrically at 595 nm. The concentration of protein in tissue homogenate was calculated using the standard curve of protein (1–10 mg/mL of bovine serum albumin in water) and expressed as µg/g tissue.

### 2.8. Histopathological Examination of Liver Tissues

Liver tissue slices were fixed in 10% paraformaldehyde and embedded in paraffin wax blocks. Sections of 5 μm thick were stained with hematoxylin & eosin (H&E) and Masson’s trichrome, then examined under light microscope for determination of pathological changes [34].

### 2.9. Statistical and Multivariate Data Analysis

All data were expressed as mean ± SD (n = 8). Statistical analysis was carried out by one-way analysis of variance (ANOVA) and the Costat software computer program. Significance values between groups were calculated at *p* < 0.05. The percentage change versus control (− or +) was calculated according to Motawi et al. [35], where the negative control was the normal healthy rats, and the positive control was the diabetic rats left untreated.

% of change = (Control mean − Treated mean)/Control mean

% of improvement = (Treated mean − Diabetic mean)/Control mean

In addition, correlation was established between investigated biological parameters and phytochemical composition based on regression analysis using Excel 2013 (Microsoft, Redmond, WA, USA). The R^2^ values were calculated for different metabolites versus % improvement in each parameter, following treatment with each sprout extract. As well, PLS analysis was performed using SIMCA software (v. 14.1, Umetrics, Umeå, Sweden).

## 3. Results and Discussion

### 3.1. Antihyperglycemic Activity

The antihyperglycemic effect was assessed through monitoring of various parameters, viz., blood glucose level, oxidative stress markers, liver enzyme function, and histopathological liver examination in comparison with STZ-diabetic rats (Group 2). The dose of trigonelline was set at 1.2 mg/kg b.wt, which was the minimum amount of trigonelline found in 15 mg lentil sprout extract, according to NMR quantification results [8], to allow for comparison between levels present naturally in sprouts.

Trigonelline was detected as a major bioactive component of potential hypoglycemic action in legume sprouts utilizing NMR and UPLC–UV–MS [8,36], and it is well reported for its antidiabetic action [37]. Consequently, the hypoglycemic action of these sprout extracts was evaluated in comparison with the trigonelline standard and glibenclamide as positive drug controls.

Post DM induction by STZ, a significant increase in the blood glucose level of diabetic rats was observed at 435%, compared to the control group (Table 1). Treatment with chickpea, lentil, fenugreek, faba bean, trigonelline, and glibenclamide standard led to a significant decrease (*p* < 0.05) in sugar levels by 23.6, 36.3, 26.6, 65.0, 29.7, and 68.1%, respectively, compared to the diabetic control group. Accordingly, improvement levels reached 126.4, 194.2, 143.1, 348.0, 159.1, and 364.3%, respectively (Table 1).

Notably, faba bean sprout recorded the most potent effect in decreasing blood glucose levels, concurrent with the highest level of antioxidant flavonoids in our previous study at 178.0 ± 7.3 mg rutin equivalent (RE)/g dried sprouts methanol extract, and the unique presence of kaempferol and its various glycosides, based on UPLC-PDA-MS analysis [7,38]. Aside from flavonoids known for antidiabetic effect, they also serve to neutralize reactive oxygen species (ROS) resulting from DM oxidative stress mitigating the incidence of organ dysfunction [39].

### 3.2. Management of Diabetes Complications

STZ exposure induced diabetes, accompanied by liver damage, as manifested by elevated levels of hepatic marker enzymes, i.e., serum AST, ALT, and ALP. STZ-induced hepatic damage is also manifested in liver histopathological changes, i.e., severe vesicular steatosis, hepatocyte ballooning, and lobular inflammation [40].

#### 3.2.1. Liver Enzymes

The effect of the various treatments on serum AST, ALT, and ALP in diabetic rats is summarized in Table 1. Results showed a significant increase in ALT level in the diabetic group by 52.70%, as compared with the control group. Treatment of diabetic rats with sprouts derived from chickpea, lentil, fenugreek, and faba bean, alongside trigonelline and glibenclamide standards, exhibited a significant decrease (*p* < 0.05) in ALT level by 15.9, 23.0, 14.6, 23.9, 20.8, and 28.3%, respectively, compared with the diabetic group. Based on that, the ALT level was improved by 24.3, 35.1, 22.3, 36.5, 31.8, and 43.2%, respectively, following these treatments. Faba bean and lentil were the most active sprouts, whereas trigonelline showed a decrease similar to that produced by chickpea and fenugreek sprouts.

Likewise, a significant increase in the AST level (Table 1) was observed in the diabetic group at 62.5%, compared with the control group. Treatment with chickpea, lentil, fenugreek, faba bean, trigonelline, and glibenclamide recorded a decrease in AST of 3.8, 12.6, 8.8, 14.8, 2.7, and 34.1%, respectively, as compared to the diabetic group. Accordingly, the improvement levels reached 6.3, 20.5, 14.3, 24.1, 4.5, and 55.4%, respectively. There was no significant difference (*p* > 0.05) between the sprout extracts and trigonelline treatment groups, whereas glibenclamide reduced AST levels almost to the normal level suggested for its superiority in that effect.

Thirdly, ALP showed a significant increase in the diabetic group, amounting to 67.5%, compared with the control group. Nevertheless, treatment with legume sprouts, including chickpea, lentil, fenugreek, faba bean, trigonelline, and glibenclamide, resulted in a significant decrease (*p* < 0.05) in ALP levels of 25.3, 23.9, 26.4, 35.7, 26.8, and 37.0%, respectively, as compared to diabetic group. The improvement levels were calculated to be at 42.3, 40.0, 44.7, 59.8, 44.9, and 62.0%, respectively, following treatment (Table 1). Faba bean and glibenclamide showed the strongest improvement in ALP levels, whereas other sprouts and trigonelline showed similar ALP decreasing activity.

It is worth mentioning that treatment with faba bean sprout extract exhibited the strongest effect in improving the levels of all investigated liver function enzymes, including ALT, AST, and ALP. Such improvement might be attributed to its richness in flavonoids, particularly kaempferol and its glycosides, which are known for their antioxidant and hepatoprotective activity [8,41].

#### 3.2.2. Lipid Profile

DM is commonly associated with dyslipidemia that can lead to an increased predisposition toward development of non-alcoholic fatty liver disease and risk of heart diseases [42,43]. Dyslipidemia is characterized by elevated levels of bad fats, i.e., cholesterol, HDL, and TG, concurrent with a decrease in HDL level. Hence, the current study further investigated the effect of various legume sprouts on the lipids profile associated with DM, presenting an added value aside from the hypoglycemic action.

**Figure 1:**
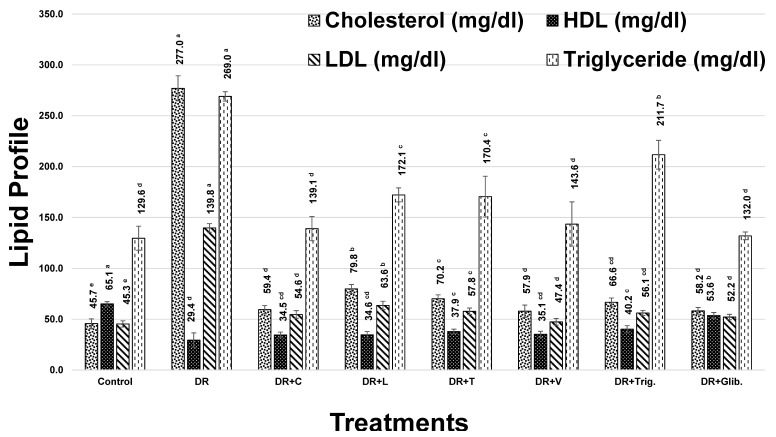
Effect of different treatments on lipid profile of diabetic rats (DR, Diabetic Rats; C, Chickpea; L, Lentil; T, Fenugreek; V, faba bean; Trig., Trigonelline; Glib., Glibenclamide). The values are represented as mean (n = 8) ± SD. Statistical analysis is carried out by one-way ANOVA where groups having the same letters (a–e) in superscripts are not significantly different, while those having different letters are significantly different at *p* < 0.05.

Cholesterol levels revealed a marked increase in diabetic rats by 506.7%, as compared to the control group, Figure 1. Treatment with chickpea, lentil, fenugreek, and faba bean sprout extracts, alongside trigonelline and glibenclamide standards, showed a significant decrease (*p* < 0.05) in cholesterol level, while faba bean and chickpea were the most active sprouts in decreasing cholesterol level by 79.1 and 78.6%, respectively. Trigonelline showed a moderate decrease of 76%, followed by fenugreek and lentil sprouts with 74.7 and 71.2, respectively, in Figure 1.

In addition, HDL-C demonstrated a significant decrease in diabetic rats of 54.8%, compared with the control group. Treatment with chickpea, lentil, fenugreek, and faba bean sprout extract, alongside trigonelline and glibenclamide, resulted in a significant increase in HDL levels (*p* < 0.05). Trigonelline and fenugreek showed the most improvement in HDL-C levels with 36.6 and 28.8, respectively (Figure 1). This effect may account for fenugreek sprout being the richest in trigonelline and 4-hydroxyisoleucine, as revealed using UPLC-MS and NMR [7,8], both known to exert an HDL-C improvement effect [44,45].

Moreover, serum LDL-C showed a significant increase in diabetic rats of 208.5%, compared with the control group. However, treatment with chickpea, lentil, fenugreek, faba bean sprouts, trigonelline, and glibenclamide standards exerted a significant decrease in LDL levels (*p* < 0.05), amounting to 60.9, 54.5, 58.6, 66.1, 59.9, and 62.7%, respectively, as compared to the diabetic group. Remarkable improvement levels were observed at 188.0, 168.2, 180.9, 203.9, 184.7, and 193.4%, respectively, with regard to the previous treatments (Figure 1). Faba bean showed the highest improvement in LDL-C among other treatments, including the standardly used drugs glibenclamide and trigonelline, presenting an added value for faba bean sprout. The reduction of LDL-C might be attributed to the flavonoids enrichment exemplified by kaempferol conjugates, which are known to induce the expression of LDL receptors [46].

Furthermore, induction of DM was associated with a significant increase in serum TG, reaching 107.6%, compared with the control group. Treatment with chickpea, lentil, fenugreek, faba bean, trigonelline, and glibenclamide showed a significant decrease in TG levels (*p* < 0.05), amounting to 48.3, 36.0, 36.7, 46.6, 21.3, and 50.6%, respectively, with improvement levels that reached 100.2, 74.8, 76.1, 96.8, 44.2, and 105.1 %, respectively, after treatment, Figure 1. Hence, these results demonstrated that chickpea, faba bean, and glibenclamide were the most active, in agreement with previous findings [47].

In conclusion, treatment with faba bean showed more or less the highest improvement in cholesterol, TG, and LDL levels, while trigonelline and fenugreek showed the highest improvement in HDL levels.

#### 3.2.3. Liver Oxidative Stress Markers

The antioxidant effect of natural products, such as flavonoids, has been reported to play a potential role aiding in DM management, including its complications, such as cardiomyopathy via alleviation of oxidative stress that is induced by elevated blood sugar levels, inflammation, and apoptosis [48,49,50]. Consequently, the antioxidant activity was assessed through the determination of SOD and GSH levels, in addition to the anti-inflammatory effect via measurement of MDA, following treatment with legume sprouts and the two drug standards, viz., glibenclamide and trigonelline. The results are summarized in Table 2.

SOD level showed a significant reduction in the liver tissue of diabetic rats, amounting to 65.8%, as compared with the control group (Table 2). However, treatment of diabetic rats with legume sprouts, trigonelline, and glibenclamide resulted in a significant increase (*p* < 0.05) of SOD activity in the order of faba bean > lentil > chickpea > fenugreek. Faba bean sprout showed the strongest elevation in SOD activity detected at 80.8%, compared to the diabetic group. Lentil showed moderate elevation of SOD activity (60.0%), followed by chickpea, fenugreek, and trigonelline, ranging from 15–27%, compared with the diabetic group.

Moreover, GSH levels in diabetic rats showed a similar significant decrease of 64.6%, compared with the control group. Diabetic rats treated with chickpea, lentil, fenugreek, faba bean, trigonelline, and glibenclamide recorded significant increases (*p* < 0.05) of 38.0, 33.2, 11.6, 71.7, 50.1, and 78.3%, respectively, as compared to the diabetic group. Hence, treatments showed improved GSH levels of 13.5, 11.7, 4.2, 25.4, 17.7, and 27.7%, respectively, revealing the same pattern of SOD, i.e., faba bean > lentil > chickpea > fenugreek (Table 2). Moreover, faba bean sprout showed the strongest improvement in GSH levels, similar to that observed by glibenclamide, which exhibited antioxidant and antimutagenic potentials against oxidative stress and organ damage [51,52]. However, trigonelline, chickpea, and lentil showed moderate GSH elevation, whereas fenugreek sprout produced the minimum GSH level improvement (Table 2).

Furthermore, significant increases in MDA levels after STZ-induced DM were detected at 173.5%, compared with the control group. Meanwhile, diabetic rats treated with chickpea, lentil, fenugreek, faba bean, trigonelline, and glibenclamide revealed significant decreases (*p* < 0.05) in MDA levels of 34.6, 48.9, 45.0, 52.8, 52.4, and 57.3%, respectively, compared with the diabetic group. Accordingly, the magnitude of improvement in MDA levels reached 94.6, 133.6, 123.0, 144.2, 143.4, and 156.6%, respectively. Both faba bean and lentil showed the highest decrease in MDA levels that could be comparable with that of the antidiabetic standard glibenclamide, Table 2.

It is worth mentioning that treatment with faba bean sprout exhibited the most potent effect in increasing SOD activity and GSH levels, in addition to decreasing MDA levels, in diabetic rats (Table 2). This effect of faba bean sprout might be explained by its rich flavonoids content and associated antioxidant activity [7,53]. The results were consistent with the previous study, which showed that faba bean sprout exhibited the highest in vitro antioxidant activity, using both DPPH and ABTS assay methods, found in strong correlation with flavonoid content [7] and in agreement with the previous report by Randhir and Shetty [54]. Such results could be further correlated with the type of flavonoid found in legume sprouts, as faba bean sprout is rich in flavonols, which have been proven previously to exert the strongest in vivo antioxidant activity among flavonoid subclasses [53].

However, the study’s limitation may be in monitoring only end products, with less insight on action mechanisms, and not testing pure isolates to prove the hypothesis, except for trigonelline. The NMR analytical platform captured the major metabolite, with less evidence of minor chemicals likely to contribute to antidiabetic efficacy and associated complications. Moreover, more clinical investigations and profiling of other legume sprouts are recommended to prove the efficacy level.

### 3.3. Histopathological Findings

**Figure 2:**
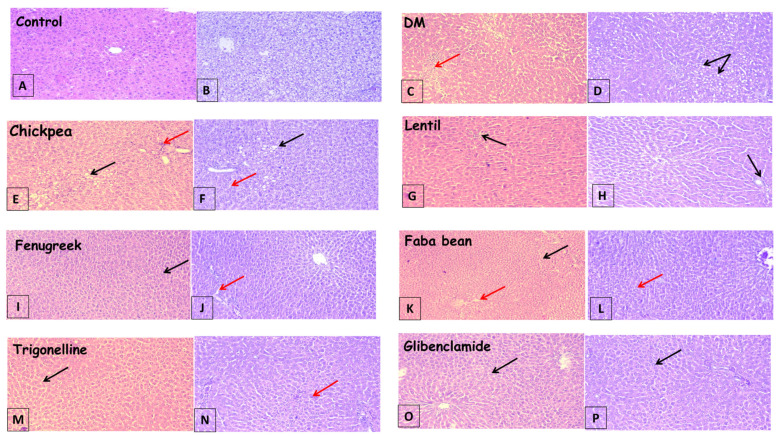
Photomicrographs of liver sections showing; (**A**,**B**): preserved lobular hepatic architecture and normal morphological appearance, (**C**,**D**): severe vesicular steatosis, hepatocyte ballooning (black arrow) and lobular inflammation (dashed arrow), (**E**,**F**): mild vesicular steatosis, hepatocyte ballooning (solid arrow) and mild inflammation (dashed arrow), (**G**,**H**): micro steatosis, hepatocyte ballooning (black arrow) with mild interlobular inflammation (dashed arrow), (**I**–**L**): intact lobular hepatic architecture, scattered few micro steatotic changes (black arrow) and minor inflammation (dashed arrow), (**M**,**N**): mild vacuolization of hepatocytes and mild ballooning of hepatocytes, and (**O**,**P**): showed more or less normal hepatic structure with minimal vacuolization. ((**A**,**C**,**E**,**G**,**I**,**K**,**M**,**O**) are stained with (**H**,**E**), while (**B**,**D**,**F**,**H**,**J**,**L**,**N**,**P**) are Masson’s trichrome, 100×).

The liver is typically susceptible to xenobiotics and/or drug-induced injury because of its central role in metabolism and its portal location within the circulatory system. Liver tissue histopathological assessment was conducted to determine the degree of architectural changes due to the different treatments and to confirm results obtained by liver function enzyme assays (Table 1). The histopathological examination of liver tissue in the current study is shown in Figure 2.

Histopathological examination of the liver in control rats revealed preserved lobular hepatic architecture and normal morphological appearance (Figure 2A,B). The histological changes in the liver injury induced by STZ revealed a high level of lobular inflammation, severe vesicular steatosis, and hepatocyte ballooning in the STZ group rats, compared with the normal histological liver (Figure 2C,D).

In contrast, all legume sprouts-treated groups showed preserved lobular hepatic architecture, normal morphological appearance, improvement in steatosis, and mild interlobular inflammation (Figure 2E–L), compared to the diabetic group. Faba bean sprout treatments showed the most improvement in liver architecture with vacuolization (Figure 2K,L) and in agreement with biochemical assays for liver functions, shown in Table 1.

In diabetic rats treated with both antidiabetic drugs, i.e., trigonelline and glibenclamide, hepatocytes degeneration, necrosis, and infiltration of inflammatory cells were all apparently ameliorated (Figure 2M,N) and (Figure 2O,P). The results showed that the treatment of diabetic rats with faba bean sprouts recorded the most hepatoprotective effect (Figure 2K,L) among treatments, which confirms the results of the liver biochemical parameters analysis and correlates to some extent with the total flavonoids content of legume sprouts [7].

### 3.4. Bioactivities in Relationship with Major Metabolites in Legume Sprouts

Following experimental results, analysis of data by chemometric tools is typically performed, based on either a supervised or unsupervised approach to aid in drug discovery. Examples include principal component analysis (PCA) and PLS. Such data analysis investigates multiple and interacting factors that help in identification and determination of potential markers specifically [55,56]. In relation to major primary and secondary metabolites quantified in our previous publications on these sprouts using MS and NMR [7,8], we attempted to use such a matrix for identification of active agents using chemometric tools. ^1^H-NMR quantification of the metabolites detected in different samples of legume sprouts’ methanol extracts is presented in the Supplementary material (Table S1). Sugars and isoflavonoids were predominant in chickpea; amino acids, choline, L-DOPA, and betaine in faba bean; in addition to trigonelline and 4-hydroxyisoleucine in fenugreek sprout extracts [7,8].

Simply, regression analysis was done as the first step to determine the regression coefficient (R^2^) by correlating the % improvement of each parameter with the major metabolites separately, where R^2^ values close to 1.0 indicate stronger relationships. Furthermore, more advanced PLS modeling was carried out investigating all interacting factors at the same time being able to identify the potential metabolite markers responsible for the antidiabetic activity in legume sprouts [57].

#### 3.4.1. Regression Analysis

Results summarizing the calculated R^2^ are listed in Table S2. They revealed that blood glucose levels showed a strong correlation with the sprouts’ flavonoid content (R^2^ = 0.85), and to a much less extent for histidine (R^2^ = 0.53), while trigonelline content failed to show a potential correlation (R^2^ = 0.32), in parallel with the moderate effect shown in the group treated with trigonelline, Table 1. These results suggested that the main antihyperglycemic action in sprouts is mediated via the inhibition of carbohydrates’ digestive enzymes by flavonoids, i.e., *α*-glucosidase, reducing metabolism and, thus, the bioavailability of sugars [13], with prevention of pancreatic cell damage by STZ-induced oxidative stress [58]. Histidine-rich supplements were reported to alleviate insulin resistance due to their anti-inflammatory activity in clinical studies [59]. Interestingly, L-DOPA content, which was detected only in faba bean sprouts, showed a strong relationship with antihyperglycemic activity (R^2^ = 0.92). The hypoglycemic effect of L-DOPA has not been shown before in previous literature. Nevertheless, this action might be mediated by the antioxidant and anti-inflammatory activities of L-DOPA [60]. Hence, flavonoid and L-DOPA contents in faba bean sprouts could account for the potential hypoglycemic effect.

The improvement percentage for the different calculated parameters, where moderate relationships with R^2^ at 0.79, 0.84, and 0.72 for L-DOPA, betaine, and total flavonoid content, respectively, for GSH, while a strong relationship was observed with histidine, with an improvement of MDA. L-DOPA, betaine, and total flavonoids were also revealed as key determinants for the improvement of ALP levels, with R^2^ values of 0.96, 0.93, and 0.99, respectively. Furthermore, L-DOPA, betaine, and total flavonoids correlated with LDL levels positively at R^2^ values of 0.69, 0.70, and 0.77, respectively. However, the effect on HDL was less obvious, Table S2.

Betaine supplements have been reported to improve liver functions, in agreement with our findings to improve SOD and GSH activities and lower TG levels [61,62]. Nevertheless, the effect of betaine levels on TG levels could not be confirmed with a weak relationship (R^2^ = 0.28). Histidine is an essential amino acid that has demonstrated beneficial health benefits for the liver through several roles, including scavenging of reactive oxygen and nitrogen species, increasing rates of MDA formations, increased GSH content, in addition to a decrease of inflammatory mediators, such as IL-6 and TNF-*α* [63,64]. Yet, the role of L-DOPA in diabetes is still not clear from previous literature, and its contribution to a liver protection effect warrants more studies in the future.

#### 3.4.2. Partial Least Square Analysis (PLS)

The PLS model was established based on the NMR fingerprint and total flavonoid content that were reported in our previous publication [8], and in correlation with the different bioactivities reported in this study. Both untargeted as NMR bins of the whole NMR region from 0–11 ppm, and targeted approaches for targeted quantified NMR peaks were attempted and compared to reveal the possible correlations between identified metabolites (X-variables) and investigated parameters (Y-variables).

- Untargeted PLS approach

**Figure 3:**
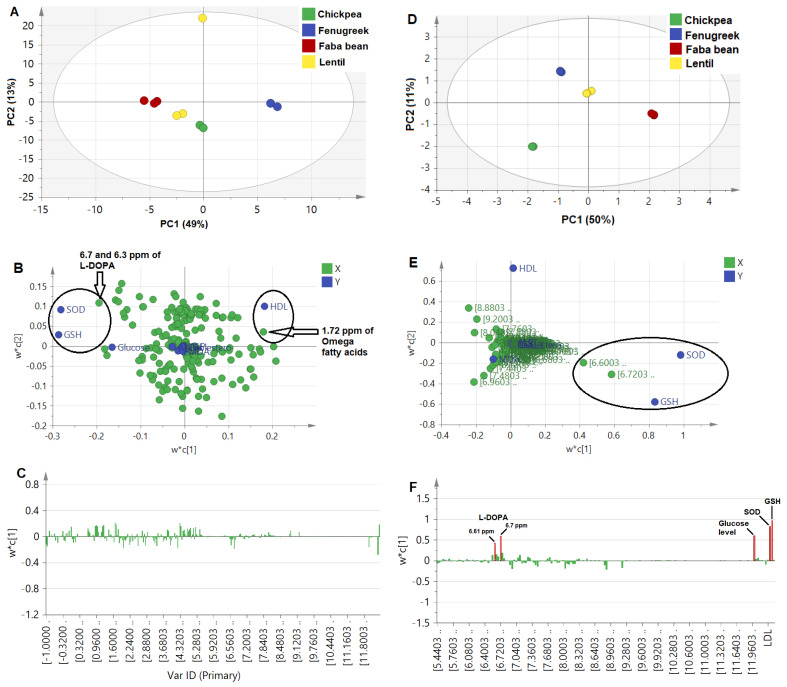
Untargeted PLS analysis for NMR spectral bins (0.04 ppm each) of the whole NMR scale (*δ*_H_ 0.0−11.0 ppm) in (**A**−**C**), in addition to aromatic region (*δ*_H_ 5.5−11.0 ppm) in (**D**−**F**). (**A**,**D**): Score plot, (**B**,**E**): Loading scatter plot, and (**C**,**F**): Loading bar plot. It should be noted that ellipses drawn do not reflect statistical significance.

Firstly, binned NMR spectra from all sprouts (*δ*_H_ 0.0–11.0 ppm) of 0.04 ppm width were modeled against the different antidiabetic activity and associated complication parameters. The score plot (Figure 3A) demonstrated the wide distance of the fenugreek sprout from all other sprouts, which could be correlated with HDL levels to the right of the loading plot (Figure 3B). In addition, faba bean sprout was found on the most left side of the score plot, correlated with GSH and SOD being rich in aromatic signals at the 6.7 and 6.3 ppm assigned for L-DOPA [8]. The corresponding loading bar plot (Figure 3C) failed to reveal a clear correlation between activity and NMR signals, likely due to the abundance of primary metabolites, i.e., sugars and organic acids, as is typical in most plant extracts, contributing less to the sprouts’ efficacy. Additionally, prediction power (Q^2^) was weak at 0.21, although of good variance coverage (R^2^ of Y-intercept = 0.83), based on the 4 calculated PCs. This model’s weakness is likely due to interference from sugar signals not related to the health effect and abundance in the sprout samples. Consequently, another model was constructed from the aromatic region (*δ*_H_ 5.5–11.0 ppm) more specific toward secondary metabolites and likely to account for the antidiabetic effect. Our previous NMR metabolomics revealed that aromatic regions provided a stronger PCA model [17], and it is first time to be examined here in the case of PLS.

The PLS model based on the aromatic region showed a higher prediction power Q^2^ of 0.43, compared to the whole NMR region model at only 0.21. The score plot in Figure 3D shows that faba bean was the most distant due to its richness in NMR signals at 6.7 and 6.3 ppm, correlating strongly with the SOD and GSH effect (Figure 3E), and in agreement with untargeted loading plot results for the whole scale (Figure 3B). In addition, the loading plot showed a positive correlation of antioxidant assays, i.e., SOD and GSH, with L-DOPA and, to a less extent, a glucose-lowering effect (Figure 3F), in contrast to previous studies that showed the neurotoxic effect of L-DOPA resulting from induced oxidative stress, except for low concentrations <30 µM [65]. The biplot overlaying score and loading plots in one figure showed a correlation of antioxidant and glucose-lowering mostly close to faba bean sprouts and correlated with L-DOPA and trigonelline to a lesser extent (Figure S1A). Additionally, the VIP score for L-DOPA signals was measured at 2, which is considered significant (Figure S1B). The relationship between the observed versus predicted effect showed the most discriminant PLS model of L-DOPA for the GSH and glucose-lowering effect (R^2^ > 0.9) and SOD (R^2^ = 0.83), Figure S1C.

Furthermore, a final model was developed to exclude the hyperglycemic effect of L-DOPA on glucose levels by removing blood glucose level parameters as the Y-variable. The bar loading plot (Figure S1D) showed a more prominent positive correlation of antioxidant assay in SOD and GSH with L-DOPA, in addition to a VIP score above 2 (Figure S1E). Confirming L-DOPA’s antioxidant effect [60] and whether it contributed to antidiabetic action needs to be further examined.

Considering that we failed to include signals from aliphatic and sugar regions, we finally attempted a third PLS model, in which total quantified metabolites expressed as µg/mg dry matter, along with the total flavonoids, were modeled along biological effects in a rather targeted approach.

- Targeted PLS approach

**Figure 4:**
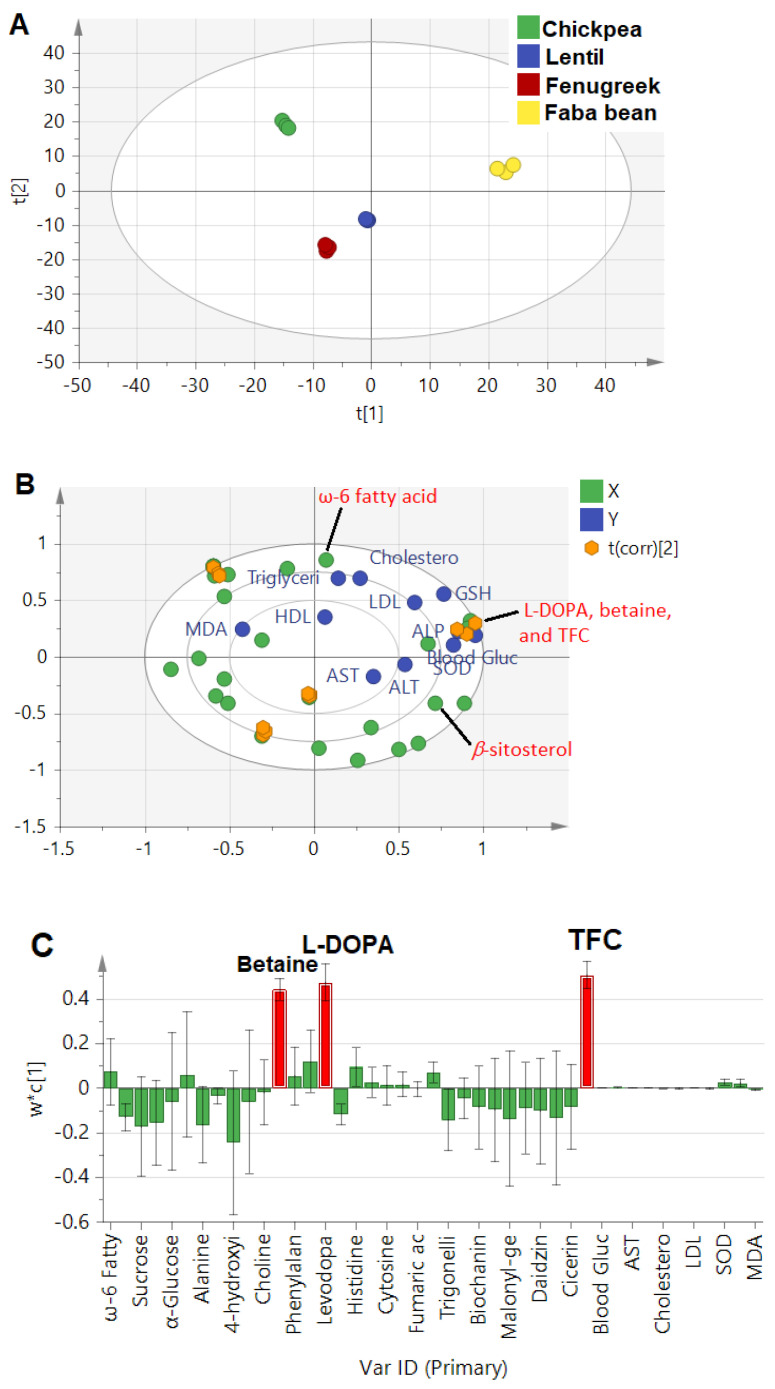
(**A**): PLS score plot of investigated legume sprouts based on quantified metabolites by NMR and total flavonoid content (TFC). (**B**): Biplot relating the quantified metabolites (X−Variables) correlated with investigated parameters (Y−Variables) related to the antidiabetic activity, and (**C**): Loading bar plot showed the positive correlation of the antidiabetic activity with identified metabolites and TFC. The t(corr) [2] sign denotes the correlation sites between X− and Y−variables.

The constructed model combining absolute quantification of metabolites and measured biological parameters showed much higher prediction power at 0.8, compared to 0.2 and 0.4 in the whole and aromatic NMR regions, respectively, that were based on an untargeted PLS approach. Such a model is expected to better pinpoint potential candidates for the prediction of metabolites mediating legume sprouts’ antidiabetic actions. The score plot (Figure 4A) revealed that faba bean was the most discriminant sample, found on the right side with positive score values from other sprout samples. The biplot (Figure 4B) revealed a strong correlation of L-DOPA, betaine, and total flavonoid content (TFC) with blood glucose, GSH, and SOD, confirming the previous results found in regression analysis (Section 3.4.1) and a further untargeted approach for L-DOPA. Additionally, the bar loading plot (Figure 4C) confirmed the prominent effect of TFC, followed by L-DOPA and betaine. Flavonoids have been well documented for their antidiabetic effect in foods and, more specifically, in legume sprouts, including extract of *Lens culinaris* and associated serum-lipid profiles [14], and *Cicer arietinum* extracts via their antioxidant and intestinal *α*-glucosidase inhibitory effects [13].

The model’s validation was performed by the goodness of fit studied through regression analysis of Y-observed versus Y-predicted values of the selected Y-variables, revealing an R^2^ close to 1 in the case of blood glucose (R^2^ = 0.97), ALP (R^2^ = 0.96), LDL (R^2^ = 0.95), SOD (R^2^ = 0.95), and GSH (R^2^ = 0.97). However, other parameters, such as ALT, TG, and MDA, showed moderate relationships at R^2^ values of 0.8, 0.81, and 0.52, respectively (Figure S2). Additionally, a permutations test was carried out to further check the model reproducibility in 100 trials. The results showed significant models, where the R^2^ values were at <0.3 and Q^2^ < 0.05 in biomarkers associated with antidiabetic activity (Figure S3).

## 4. Conclusions and Future Perspectives

This study represents the first biochemometric approach to assess the antidiabetic activity of four major legume sprouts and their mitigation against diabetic-associated complications, as determined using NMR to reveal active agents in sprout extracts. The application of the targeted versus untargeted PLS regression models aided in revealing active agents to mediate a decrease in serum glucose level, particularly for L-DOPA. Faba bean sprout showed the strongest effect, compared to glibenclamide, among other sprouts, posing its future inclusion in nutraceuticals for diabetic patients. This effect is likely attributed to its richness in flavonoids, alongside high levels of L-DOPA absent in all the other sprouts. Other nitrogenous compounds, i.e., betaine and histidine, appeared to be more related to the hepatoprotective and antihyperlipidemic effects. Moreover, flavonoids played a more discriminatory role in sprouts´ antidiabetic action than trigonelline, although the latter is known as an antidiabetic agent. The antioxidant effect was more associated with L-DOPA, while HDL was more correlated to omega fatty acids (e.g., ω6-fatty acids). Conclusively, our results pinpoint the benefit of sprouts consumption for improving decreases of insulin resistance, scavenging ROS, and anti-inflammatory activity. However, the current study suffers from some limitations, such as monitoring of only end products and not testing pure isolated metabolites, except in the case of trigonelline.

Further studies are recommended for revealing action mechanisms either for the crude extracts or pure isolated metabolites, as well as more clinical investigations and profiling of other legume sprouts to aid in identifying more active agents. Moreover, examining changes in the levels of these chemicals with sprouting can identify the best stages for their harvest. Investigation of the possible synergistic effects of these sprouts and standard antidiabetic drugs should be examined to help provide the most efficacious regimens and identify any potential interactions.

## Figures and Tables

**Table 1:** Effect of different treatments on serum alanine aminotransferase (ALT), aspartate aminotransferase (AST), and alkaline phosphatase (ALP) of diabetic rats (n = 8).

Groups	Glucose Level		ALT		AST		ALP	
	Glucose (mg/dL)	% Improvement	ALT (Unit/L)	% Improvement	AST (Unit/L)	% Improvement	ALP (Unit/L)	% Improvement
Control	74.6 ^e^ ± 2.4	---	1.5 ^c^ ± 0.1	---	2.2 ^b^ ± 0.2	---	258.5 ^d^ ± 11.0	---
DR	399.2 ^a^ ± 30.9	---	2.3 ^a^ ± 0.3	---	3.6 ^a^ ± 0.3	---	433.2 ^a^ ± 16.8	---
DR + C	304.9 ^b^ ± 36.4	126.5	1.9 ^b^ ± 0.1	24.3	3.5 ^a^ ± 0.4	6.3	323.8 ^b^ ± 5.5	42.3
DR + L	254.4 ^c^ ± 26.6	194.2	1.7 ^bc^ ± 0.1	35.1	3.2 ^a^ ±0.6	20.5	329.7 ^b^ ± 4.4	40.0
DR + F	292.6 ^bc^ ± 25.4	143.1	1.9 ^b^ ± 0.2	22.3	3.3 ^a^ ± 0.2	14.3	318.9 ^b^ ± 8.9	44.6
DR + V	139.6 ^d^ ± 11.4	348.0	1.7 ^bc^±0.1	36.5	3.1 ^a^ ± 0.1	24.1	278.6 ^c^ ± 9.2	59.8
DR + Trig.	280.5 ^bc^ ± 32.0	159.1	1.8 ^b^ ± 0.2	31.8	3.5 ^a^ ± 0.4	4.5	317.1 ^b^ ± 3.3	44.9
DR + Glib.	127.4 ^d^ ± 2.5	364.3	1.6 ^bc^ ± 0.11	43.2	2.4 ^b^ ± 0.2	55.4	273.0 ^c^ ± 21.3	62.0

DR, Diabetic Rats; C, Chickpea; L, Lentil; F, Fenugreek; V, Faba bean; Trig., Trigonelline; Glib., Glibenclamide. Data are expressed as mean ± SD of eight rats in each group. Statistical analysis is carried out using one-way ANOVA where groups having the same letters (a–e) in superscripts are not significantly different, while those having different letters are significantly different at *p* < 0.05.

**Table 2:** Effect of different treatments on liver superoxide dismutase (SOD) activity, liver glutathione (GSH) level, and liver malondialdehyde (MDA) of diabetic rats (n = 8).

Group	SOD		GSH		MDA	
	SOD Level (µg/mg Protein)	% Improvement	GSH Level (µg/g Tissue)	% Improvement	MDA (µmol/mg Protein)	% Improvement
Control	28.4 ^a^ ± 1.4	---	26.0 ^a^ ± 2.0	---	1.1 ^d^ ± 0.2	---
DR	9.7 ^e^ ± 0.8	---	9.2 ^d^ ± 0.7	---	3.1 ^a^ ± 0.5	---
DR + C	12.4 ^d^ ± 1.6	9.4	12.7 ^c^ ± 1.1	13.5	2.0 ^b^ ± 0.2	94.6
DR + L	15.5 ^c^ ± 1.1	20.5	12.3 ^c^ ± 0.7	11.7	1.6 ^cd^ ± 0.2	133.6
DR + F	11.8 ^d^ ± 1.0	7.5	10.3 ^d^ ± 0.6	4.2	1.7 ^c^ ± 0.7	123.0
DR + V	17.5 ^b^ ± 1.7	27.6	15.8 ^b^ ± 0.6	25.4	1.5 ^cd^ ± 0.2	144.2
DR + Trig.	11.2 ^de^ ± 1.0	5.3	13.8 ^c^ ± 0.6	17.7	1.5 ^cd^ ± 0.2	143.4
DR + Glib.	14.7 ^c^ ± 0.7	17.5	16.4 ^b^ ± 1.6	27.7	1.3 ^cd^ ± 0.2	156.6

DR, Diabetic Rats; C, Chickpea; L, Lentil; T, Fenugreek; V, Faba bean; Trig., Trigonelline; Glib., Glibenclamide. Data are expressed as mean ± SD of eight rats in each group. Statistical analysis is carried out by one-way ANOVA where groups having the same letters (a–e) in superscripts are not significantly different, while those having different letters are significantly different at *p* < 0.05.

## Data Availability

The data presented in this study are available in article and supplementary materials.

## Supplementary Materials

The following supporting information can be downloaded at: https://www.mdpi.com/article/10.3390/metabo13010063/s1, Figure S1: A: Biplot, B: VIP score plot, and C: Observed versus predicted effect for GSH, SOD, and glucose level, showing correlation of antioxidant and glucose lowering effects in relation to binned metabolites by NMR in aromatic region (*δ*_H_ 5.5–11.0 ppm). D: Loading bar plot and E: VIP score after exclusion of glucose level; Figure S2: Y-observed versus Y-predicted plot showing the constructed model’s goodness of fit based on metabolites quantified by NMR in legume sprouts against values of various biomarkers used for evaluation of antidiabetic effect; Figure S3: Permutation tests (n = 100) of the various antidiabetic biomarkers modelled for phytochemicals identified by NMR in legume sprouts; Table S1: ^1^H-NMR quantification of major primary and secondary metabolites in different samples of legume sprouts methanol extracts. Values are expressed as μg/mg dry powder ± S.D (n = 3). Chemical shifts used for metabolite quantification were determined in methanol-d6 and expressed as relative values to HMDS (0.94 mM final concentration) Statistical analysis is carried out by one-way analysis of variance (ANOVA) where unshared letters between groups are the significance value at *p* < 0.05; Table S2: Summarized regression coefficient (R^2^) values calculated for major legume sprout metabolites against investigated parameters.

## References

[1] Vatanparast M., Powell A., Doyle J.J., Egan A.N. (2018). Targeting legume loci: A comparison of three methods for target enrichment bait design in Leguminosae phylogenomics. Appl. Plant Sci..

[2] Sutjaritjai N., Wangpakapattanawong P., Balslev H., Inta A. (2019). Traditional uses of Leguminosae among the Karen in Thailand. Plants.

[3] Tungmunnithum D., Drouet S., Lorenzo J.M., Hano C. (2021). Characterization of bioactive phenolics and antioxidant capacity of edible bean extracts of 50 Fabaceae populations grown in Thailand. Foods.

[4] Khrisanapant P., Kebede B., Leong S.Y., Oey I. (2019). A comprehensive characterisation of volatile and fatty acid profiles of legume seeds. Foods.

[5] Devi C., Kushwaha A., Kumar A. (2015). Sprouting characteristics and associated changes in nutritional composition of cowpea (*Vigna unguiculata*). J. Food Sci. Technol..

[6] Mohd Ali N., Mohd Yusof H., Long K., Yeap S.K., Ho W.Y., Beh B.K., Koh S.P., Abdullah M.P., Alitheen N.B. (2013). Antioxidant and hepatoprotective effect of aqueous extract of germinated and fermented mung bean on ethanol-mediated liver damage. BioMed Res. Int..

[7] Farag M., Sharaf El-Din M., Aboul-Fotouh Selim M., Owis A., Abouzid S. (2021). Mass spectrometry-based metabolites profiling of nutrients and anti-nutrients in major legume sprouts. Food Biosci..

[8] Farag M.A., Sharaf El-Din M.G., Selim M.A., Owis A.I., Abouzid S.F., Porzel A., Wessjohann L.A., Otify A. (2021). Nuclear magnetic resonance metabolomics approach for the analysis of major legume sprouts coupled to chemometrics. Molecules.

[9] Vijayakumar M.V., Singh S., Chhipa R.R., Bhat M.K. (2005). The hypoglycaemic activity of fenugreek seed extract is mediated through the stimulation of an insulin signalling pathway. Br. J. Pharmacol..

[10] Alberti K.G.M.M., Zimmet P.Z. (1998). Definition, diagnosis and classification of diabetes mellitus and its complications. Part 1: Diagnosis and classification of diabetes mellitus. Provisional report of a WHO consultation. Diabet. Med..

[11] Yazdi H.B., Hojati V., Shiravi A., Hosseinian S., Vaezi G., Hadjzadeh M.A. (2019). Liver Dysfunction and Oxidative Stress in Streptozotocin-Induced Diabetic Rats: Protective Role of Artemisia Turanica. J. Pharmacopuncture.

[12] Mertens J., De Block C., Spinhoven M., Driessen A., Francque S.M., Kwanten W.J. (2021). Hepatopathy associated with type 1 diabetes: Distinguishing non-alcoholic fatty liver disease From glycogenic hepatopathy. Front. Pharmacol..

[13] Tiwari A.K., Sahana C., Zehra A., Madhusudana K., Kumar D.A., Agawane S.B. (2013). Mitigation of starch-induced postprandial glycemic spikes in rats by antioxidants-rich extract of *Cicer arietinum* Linn. seeds and sprouts. J. Pharm. Bioallied Sci..

[14] Tefera M.M., Altaye B.M., Yimer E.M., Berhe D.F., Tadesse Bekele S. (2020). Antidiabetic effect of germinated *Lens culinaris* Medik seed extract in streptozotocin-induced diabetic mice. J. Exp. Pharmacol..

[15] Zhou J., Zhou S., Zeng S. (2013). Experimental diabetes treated with trigonelline: Effect on *β* cell and pancreatic oxidative parameters. Fundam. Clin. Pharmacol..

[16] Lv Q., Yang Y., Zhao Y., Gu D., He D., Yili A., Ma Q., Cheng Z., Gao Y., Aisa H.A. (2009). Comparative study on separation and purification of isoflavones from the seeds and sprouts of chickpea by high-speed countercurrent chromatography. J. Liq. Chrom. Relat. Tech..

[17] Farag M.A., Porzel A., Wessjohann L.A. (2012). Comparative metabolite profiling and fingerprinting of medicinal licorice roots using a multiplex approach of GC-MS, LC-MS and 1D NMR techniques. Phytochemistry.

[18] Farag M.A., Porzel A., Schmidt J., Wessjohann L.A. (2012). Metabolite profiling and fingerprinting of commercial cultivars of *Humulus lupulus* L. (hop): A comparison of MS and NMR methods in metabolomics. Metabolomics.

[19] Punithavathi V., Anuthama R., Prince P. (2008). Combined treatment with naringin and vitamin C ameliorates streptozotocin-induced diabetes in male Wistar rats. J. Appl. Toxicol..

[20] Bhandari U., Pillai K. (2005). Effect of ethanolic extract of *Zingiber officinale* on dyslipidaemia in diabetic rats. J. Ethnopharmacol..

[21] Vijayakumar M.V., Bhat M.K. (2008). Hypoglycemic effect of a novel dialysed fenugreek seeds extract is sustainable and is mediated, in part, by the activation of hepatic enzymes. Phytother. Res..

[22] Cheng D., Liang B., Li Y. (2012). Antihyperglycemic effect of *Ginkgo biloba* extract in streptozotocin-induced diabetes in rats. BioMed Res. Int..

[23] Cronin D., Smith S. (1979). A simple and rapid procedure for the analysis of reducing, total and individual sugars in potatoes. Potato Res..

[24] Reitman S., Frankel S. (1957). A colorimetric method for the determination of serum glutamic oxalacetic and glutamic pyruvic transaminases. Am. J. Clin. Pathol..

[25] Thomas L. (1998). Clinical Laboratory Diagnostics: Use and Assessment of Clinical Laboratory Results.

[26] Meiattini F., Prencipe L., Bardelli F., Giannini G., Tarli P. (1978). The 4-hydroxybenzoate/4-aminophenazone chromogenic system used in the enzymic determination of serum cholesterol. Clin. Chem..

[27] Burstein M., Scholnick H., Morfin R. (1970). Rapid method for the isolation of lipoproteins from human serum by precipitation with polyanions. J. Lipid Res..

[28] Assmann G., Jabs H.-U., Kohnert U., Nolte W., Schriewer H. (1984). LDL-cholesterol determination in blood serum following precipitation of LDL with polyvinylsulfate. Clin. Chim. Acta.

[29] Fossati P., Prencipe L. (1982). Serum triglycerides determined colorimetrically with an enzyme that produces hydrogen peroxide. Clin. Chem..

[30] Nishikimi M., Rao N.A., Yagi K. (1972). The occurrence of superoxide anion in the reaction of reduced phenazine methosulfate and molecular oxygen. Biochem. Biophys. Res. Commun..

[31] Moron M.S., Depierre J.W., Mannervik B. (1979). Levels of glutathione, glutathione reductase and glutathione S-transferase activities in rat lung and liver. Biochim. Biophys. Acta (BBA)-Gen. Subj..

[32] Buege J.A., Aust S.D. (1978). Microsomal lipid peroxidation. Methods Enzymol..

[33] Bradford M.M. (1976). A rapid and sensitive method for the quantitation of microgram quantities of protein utilizing the principle of protein-dye binding. Anal. Biochem..

[34] Hirsch C., Zouain C., Alves J., Goes A. (1997). Induction of protective immunity and modulation of granulomatous hypersensitivity in mice using PIII, an anionic fraction of Schistosoma mansoni adult worm. Parasitology.

[35] Motawi T.K., Darwish H.A., Hamed M.A., El-Rigal N.S., Naser A.F.A. (2017). A Therapeutic insight of niacin and coenzyme Q10 against diabetic encephalopathy in rats. Mol. Neurobiol..

[36] Farag M.A., Porzel A., Wessjohann L.A. (2015). Unraveling the active hypoglycemic agent trigonelline in *Balanites aegyptiaca* date fruit using metabolite fingerprinting by NMR. J. Pharm. Biomed. Anal..

[37] Subramanian S.P., Prasath G.S. (2014). Antidiabetic and antidyslipidemic nature of trigonelline, a major alkaloid of fenugreek seeds studied in high-fat-fed and low-dose streptozotocin-induced experimental diabetic rats. Biomed. Prev. Nutr..

[38] Yang L., Gao Y., Bajpai V.K., El-Kammar H.A., Simal-Gandara J., Cao H., Cheng K.W., Wang M., Arroo R.R.J., Zou L. (2021). Advance toward isolation, extraction, metabolism and health benefits of kaempferol, a major dietary flavonoid with future perspectives. Crit. Rev. Food Sci. Nutr..

[39] Gupta R.K., Patel A.K., Shah N., Chaudhary A.K., Jha U.K., Yadav U.C., Gupta P.K., Pakuwal U. (2014). Oxidative stress and antioxidants in disease and cancer: A review. Asian Pac. J. Cancer Prev..

[40] Safhi M.M., Alam M.F., Sivakumar S.M., Anwer T. (2019). Hepatoprotective potential of *Sargassum muticum* against STZ-induced diabetic liver damage in wistar rats by inhibiting cytokines and the apoptosis pathway. Anal. Cell. Pathol..

[41] Wang Y., Tang C., Zhang H. (2015). Hepatoprotective effects of kaempferol 3-O-rutinoside and kaempferol 3-O-glucoside from Carthamus tinctorius L. on CCl(4)-induced oxidative liver injury in mice. J. Food Drug Anal..

[42] Bhowmik B., Siddiquee T., Mujumder A., Afsana F., Ahmed T., Mdala I.A., do V Moreira N.C., Khan A.K.A., Hussain A., Holmboe-Ottesen G. (2018). Serum lipid profile and its association with diabetes and prediabetes in a rural Bangladeshi population. Int. J. Environ. Res. Public Health.

[43] Farid M.M., Aboul Naser A.F., Salem M.M., Ahmed Y.R., Emam M., Hamed M.A. (2022). Chemical compositions of Commiphora opobalsamum stem bark to alleviate liver complications in streptozotocin-induced diabetes in rats: Role of oxidative stress and DNA damage. Biomarkers.

[44] Zhang D.-F., Zhang F., Zhang J., Zhang R.-M., Li R. (2015). Protection effect of trigonelline on liver of rats with non-alcoholic fatty liver diseases. Asian Pac. J. Trop. Med..

[45] Ibarra A., He K., Bai N., Bily A., Roller M., Coussaert A., Provost N., Ripoll C. (2008). Fenugreek extract rich in 4-hydroxyisoleucine and trigonelline activates PPARα and inhibits LDL oxidation: Key mechanisms in controlling the metabolic syndrome. Nat. Prod. Commun..

[46] Ochiai A., Miyata S., Iwase M., Shimizu M., Inoue J., Sato R. (2016). Kaempferol stimulates gene expression of low-density lipoprotein receptor through activation of Sp1 in cultured hepatocytes. Sci. Rep..

[47] Skrapari I., Perrea D., Ioannidis I., Karabina S.A., Elisaf M., Tselepis A.D., Karagiannacos P., Katsilambros N. (2001). Glibenclamide improves postprandial hypertriglyceridaemia in type 2 diabetic patients by reducing chylomicrons but not the very low-density lipoprotein subfraction levels. Diabet. Med..

[48] Sarian M.N., Ahmed Q.U., Mat So’ad S.Z., Alhassan A.M., Murugesu S., Perumal V., Syed Mohamad S.N.A., Khatib A., Latip J. (2017). Antioxidant and antidiabetic effects of flavonoids: A structure-activity relationship based study. BioMed Res. Int..

[49] Jubaidi F.F., Zainalabidin S., Taib I.S., Hamid Z.A., Budin S.B. (2021). The potential role of flavonoids in ameliorating diabetic cardiomyopathy via alleviation of cardiac oxidative stress, inflammation and apoptosis. Int. J. Mol. Sci..

[50] Unuofin J.O., Lebelo S.L. (2020). Antioxidant effects and mechanisms of medicinal plants and their bioactive compounds for the prevention and treatment of type 2 diabetes: An updated review. Oxid. Med. Cell. Longev..

[51] Erejuwa O.O., Sulaiman S.A., Wahab M.S., Salam S.K., Salleh M.S., Gurtu S. (2010). Antioxidant protective effect of glibenclamide and metformin in combination with honey in pancreas of streptozotocin-induced diabetic rats. Int. J. Mol. Sci..

[52] Rabbani S.I., Devi K., Khanam S. (2010). Protective role of glibenclamide against nicotinamide-streptozotocin induced nuclear damage in diabetic Wistar rats. J. Pharmacol. Pharmacother..

[53] Zang Y., Zhang D., Yu C., Jin C., Igarashi K. (2017). Antioxidant and hepatoprotective activity of kaempferol 3-O-β-d-(2,6-di-O-α-l-rhamnopyranosyl)galactopyronoside against carbon tetrachloride-induced liver injury in mice. Food Sci. Biotechnol..

[54] Randhir R., Shetty K.J.P.B. (2004). Microwave-induced stimulation of L-DOPA, phenolics and antioxidant activity in fava bean (*Vicia faba*) for Parkinson’s diet. Process Biochem..

[55] Farag M.A., Khaled S.E., El Gingeehy Z., Shamma S.N., Zayed A. (2022). Comparative metabolite profiling and fingerprinting of medicinal cinnamon bark and its commercial preparations via a multiplex approach of GC-MS, UV, and NMR techniques. Metabolites.

[56] Mehring A., Zayed A., Salem M.A., Alseekh S., Fernie A.R., Ulber R. (2022). Time-dependent behaviour of methyl jasmonate elicited cell suspension cultures of *Ocimum* species via untargeted mass spectrometry-based metabolomics. Ind. Crops Prod..

[57] Khaled S.E., Hashem F.A.M., Shabana M.H., Hammam A.-M.M., Madboli A.N.A., Al-Mahdy D.A., Farag M.A. (2019). A biochemometric approach for the assessment of Phyllanthus emblica female fertility effects as determined via UPLC-ESI-qTOF-MS and GC-MS. Food Funct..

[58] Coskun O., Kanter M., Korkmaz A., Oter S. (2005). Quercetin, a flavonoid antioxidant, prevents and protects streptozotocin-induced oxidative stress and *β*-cell damage in rat pancreas. Pharmacol. Res..

[59] Feng R.N., Niu Y.C., Sun X.W., Li Q., Zhao C., Wang C., Guo F.C., Sun C.H., Li Y. (2013). Histidine supplementation improves insulin resistance through suppressed inflammation in obese women with the metabolic syndrome: A randomised controlled trial. Diabetologia.

[60] Aware C., Patil R., Gaikwad S., Yadav S., Bapat V., Jadhav J. (2017). Evaluation of l-dopa, proximate composition with in vitro anti-inflammatory and antioxidant activity of *Mucuna macrocarpa* beans: A future drug for Parkinson treatment. Asian Pac. J. Trop. Biomed..

[61] Arumugam M.K., Paal M.C., Donohue T.M., Ganesan M., Osna N.A., Kharbanda K.K. (2021). Beneficial effects of betaine: A comprehensive review. Biology.

[62] Wang L., Chen L., Tan Y., Wei J., Chang Y., Jin T., Zhu H. (2013). Betaine supplement alleviates hepatic triglyceride accumulation of apolipoprotein E deficient mice via reducing methylation of peroxisomal proliferator-activated receptor alpha promoter. Lipids Health Dis..

[63] Holeček M. (2020). Histidine in health and disease: Metabolism, physiological importance, and use as a supplement. Nutrients.

[64] Liu W.-H., Liu T.-C., Yin M.-C. (2008). Beneficial effects of histidine and carnosine on ethanol-induced chronic liver injury. Food Chem. Toxicol..

[65] Zhong S.Y., Chen Y.X., Fang M., Zhu X.L., Zhao Y.X., Liu X.Y. (2014). Low-dose levodopa protects nerve cells from oxidative stress and up-regulates expression of pCREB and CD39. PLoS ONE.

