# Supplementary material for: Comparative Insights into Four Major Legume Sprouts Efficacies for Diabetes Management and Its Complications: Untargeted versus Targeted NMR Biochemometrics Approach
Source: Metabolites. 2022 Dec 31;13(1):63. doi: 10.3390/metabo13010063 (PMC9866814; doi:10.3390/metabo13010063)
Supplement: Supplementary file 1 [file metabolites-13-00063-s001.zip › metabolites-2067686-supplementary.pdf]

Supplementary Tables

Table S1: ^{1}H-NMR quantification of major primary and secondary metabolites in different samples of legume sprouts methanol extracts. Values are expressed as µg/mg dry powder ± S.D (n=3). Chemical shifts used for metabolite quantification were determined in methanol-d_6 and expressed as relative values to HMDS (0.94 mM final concentration) Statistical analysis is carried out by one-way analysis of variance (ANOVA) where unshared letters between groups are the significance value at p<0.05.

ID	Compound	Amount µg/mg dry matter			
		Chickpea	Lentil	Fenugreek	Faba bean
1	ω-6 Fatty acid	51.19 ± 4.58^{a}	41.32 ± 4.26^{bc}	38.16 ± 1.78^{c}	47.77 ± 4.12^{ab}
2	ω-3 Fatty acid	20.12 ± 1.76^{a}	11.96 ± 0.85^{b}	21.69 ± 0.49^{a}	13.00 ± 1.30^{b}
3	Sucrose	239.82 ± 6.98^{a}	144.67 ± 5.87^{c}	178.74 ± 3.39^{b}	172.96 ± 7.80^{b}
4	Fructose	148.39 ± 3.67^{a}	82.91 ± 2.50^{d}	103.72 ± 1.55^{b}	95.60 ± 3.81^{c}
5	*α*-Glucose	36.89 ± 5.11^{c}	73.43 ± 5.28^{b}	94.92 ± 1.76^{a}	71.06 ± 7.58^{b}
6	*β*-Glucose	43.15 ± 3.33^{c}	76.31 ± 5.11^{b}	89.00 ± 7.70^{a}	81.72 ± 4.15^{ab}
7	Alanine	31.46 ± 1.88^{b}	23.51 ± 2.51^{c}	44.59 ± 1.88^{a}	25.01 ± 1.19^{c}
8	Valine	12.61 ± 0.63^{b}	14.59 ± 0.95^{a}	12.57 ± 0.75^{b}	10.26 ± 0.83^{c}
10	4-Hydroxyisoleucine	0.0 ± 0.0^{b}	0.0 ± 0.0^{b}	51.13 ± 3.53^{a}	0.0 ± 0.0^{b}
11	Asparagine	61.05 ± 4.51^{b}	73.46 ± 8.09^{b}	93.43 ± 4.29^{a}	72.71 ± 9.68^{b}
12	Choline	19.06 ± 0.54^{a}	16.91 ± 1.09^{b}	9.06 ± 0.20^{c}	9.94 ± 0.95^{c}
13	Betaine	12.98 ± 0.62^{b}	10.06 ± 0.87^{bc}	5.06 ± 1.10^{c}	109.16 ± 5.49^{a}
15	Phenylalanine	0.0 ± 0.0^{b}	8.61 ± 0.62^{a}	8.69 ± 0.94^{a}	9.07 ± 1.45^{a}
16	Tyrosine	0.0 ± 0.0^{c}	8.59 ± 0.53^{b}	8.93 ± 0.68^{b}	15.57 ± 2.69^{a}
17	L-Dopa	0.0 ± 0.0^{b}	0.0 ± 0.0^{b}	0.0 ± 0.0^{b}	112.40 ± 13.16^{a}
18	Tryptophan	24.16 ± 5.02^{a}	22.82 ± 3.70^{a}	22.05 ± 2.32^{a}	10.36 ± 2.99^{b}
19	Histidine	4.23 ± 0.25^{c}	11.22 ± 1.77^{a}	7.43 ± 1.91^{b}	11.07 ± 1.77^{a}
20	Cytosine	9.39 ± 1.95^{a}	6.16 ± 1.30^{b}	5.53 ± 0.72^{b}	7.30 ± 1.45^{ab}
22	Acetic acid	0.0 ± 0.0^{b}	10.51 ± 0.46^{a}	0.0 ± 0.0^{b}	0.0 ± 0.0^{b}
23	Fumaric acid	2.18 ± 0.19^{c}	2.51 ± 0.25^{bc}	3.11 ± 0.16^{a}	2.84 ± 0.34^{ab}
24	*β*-Sitosterol	8.95 ± 0.67^{b}	10.12 ± 0.78^{ab}	8.56 ± 1.03^{b}	10.77 ± 0.90^{a}
25	Trigonelline	18.03 ± 0.97^{b}	8.11 ± 1.02^{d}	24.73 ± 1.02^{a}	11.59 ± 1.34^{c}
26	Biochanin A	32.04 ± 2.12^{a}	0.0 ± 0.0^{b}	0.0 ± 0.0^{b}	0.0 ± 0.0^{b}
27	Genistin	43.86 ± 4.87^{a}	0.0 ± 0.0^{b}	0.0 ± 0.0^{b}	0.0 ± 0.0^{b}
28	Malonyl-genistin	78.88 ± 1.46^{a}	0.0 ± 0.0^{b}	0.0 ± 0.0^{b}	0.0 ± 0.0^{b}
29	Formononetin	35.52 ± 2.00^{a}	0.0 ± 0.0^{b}	0.0 ± 0.0^{b}	0.0 ± 0.0^{b}
30	Daidzin	49.27 ± 3.10^{a}	0.0 ± 0.0^{b}	0.0 ± 0.0^{b}	0.0 ± 0.0^{b}
31	Malonyl-daidzin	80.22 ± 3.56^{a}	0.0 ± 0.0^{b}	0.0 ± 0.0^{b}	0.0 ± 0.0^{b}
32	Cicerin	33.19 ± 2.84^{a}	0.0 ± 0.0^{b}	0.0 ± 0.0^{b}	0.0 ± 0.0^{b}

Table S2: Summarized regression coefficient (R^2) values calculated for major legume sprout metabolites against investigated parameters.

Major metabolites	Blood glucose	GSH	MDA	ALT	AST	ALP	SOD	Cholesterol	LDL	HDL	TG
**Total flavonoid content**	0.85	0.72	0.35	0.3	0.37	0.99	0.52	0.41	0.77	0.0	0.26
**Choline**	0.22	0	0.38	0	0.27	0.4	0.03	0.02	0.16	0.55	0.05
***β*-Sitosterol**	0.8	0.71	0.55	0.96	0.69	0.38	0.99	0	0.12	0.32	0.04
**Trigonelline**	0.32	0.38	0.25	0.86	0.38	0.03	0.69	0.05	0	0.63	0
**ω6-Fatty acid**	0.04	0.43	0.17	0.02	0.09	0.09	0.04	0.6	0.37	0.5	0.92
**L-DOPA**	0.92	0.79	0.4	0.4	0.44	0.96	0.63	0.34	0.7	0.03	0.24
**Betaine**	0.91	0.84	0.37	0.42	0.41	0.94	0.65	0.36	0.71	0.06	0.28
**Histidine**	0.54	0.16	0.92	0.75	0.96	0.17	0.7	0.16	0	0.01	0.15
**4-Hydroxyisoleucine**	0.16	0.52	0	0.44	0.03	0.02	0.38	0.05	0.04	0.97	0.29
**Alanine**	0.32	0.53	0.13	0.77	0.24	0.05	0.66	0	0.01	0.8	0.08
**Cystosine**	0.01	0.17	0.42	0.01	0.31	0	0	0.48	0.19	0.41	0.8
**Asparagine**	0.01	0.31	0.15	0.08	0.06	0	0.07	0.16	0.05	0.87	0.52

Supplementary Figures

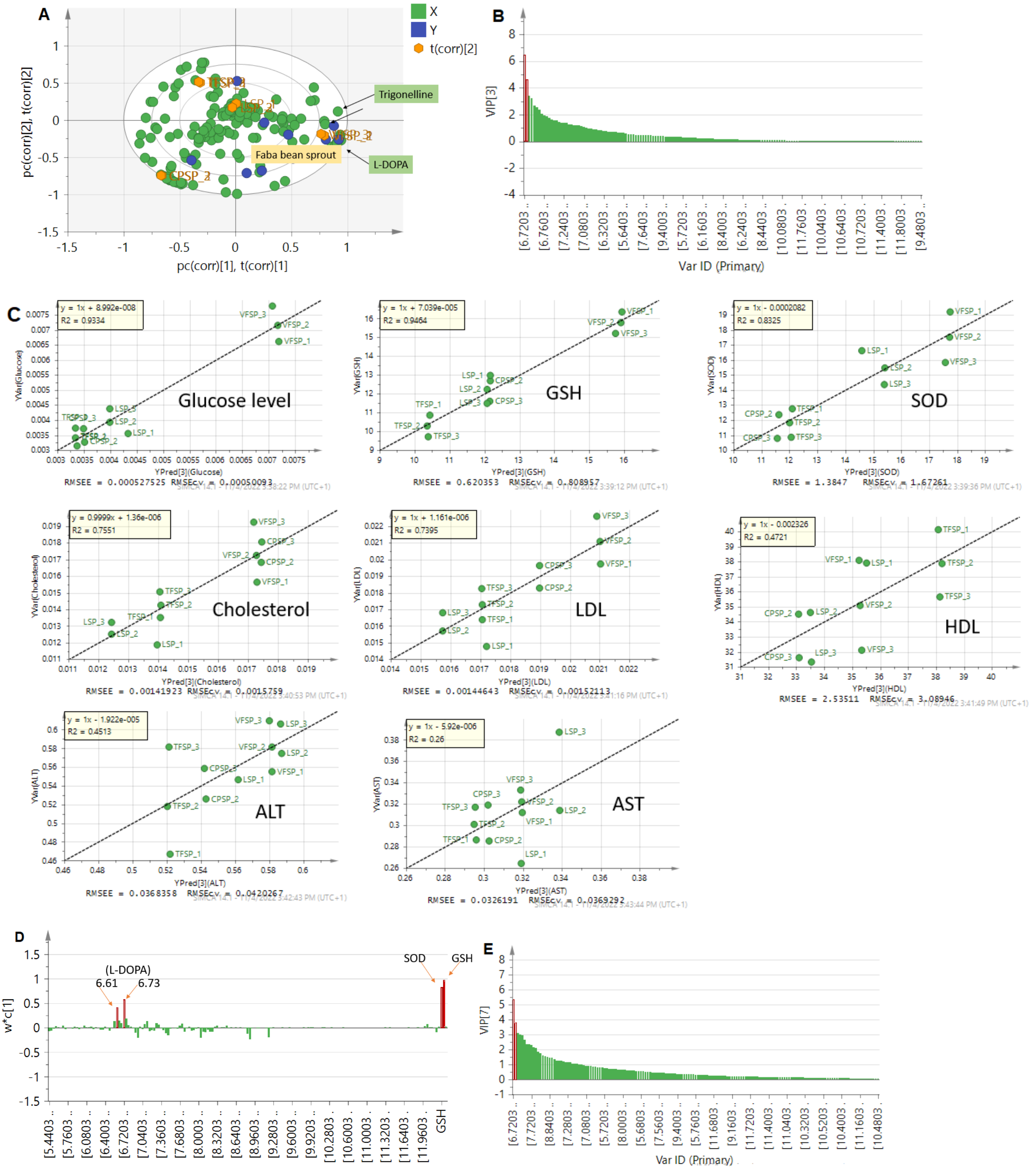

Figure S1. A: Biplot, **B:** VIP score plot, and **C:** Observed versus predicted effect for GSH, SOD, and glucose level, showing correlation of antioxidant and glucose lowering effects in relation to binned metabolites by NMR in aromatic region (δ_H 5.5-11.0 ppm). **D:** Loading bar plot and **E:** VIP score after exclusion of glucose level.

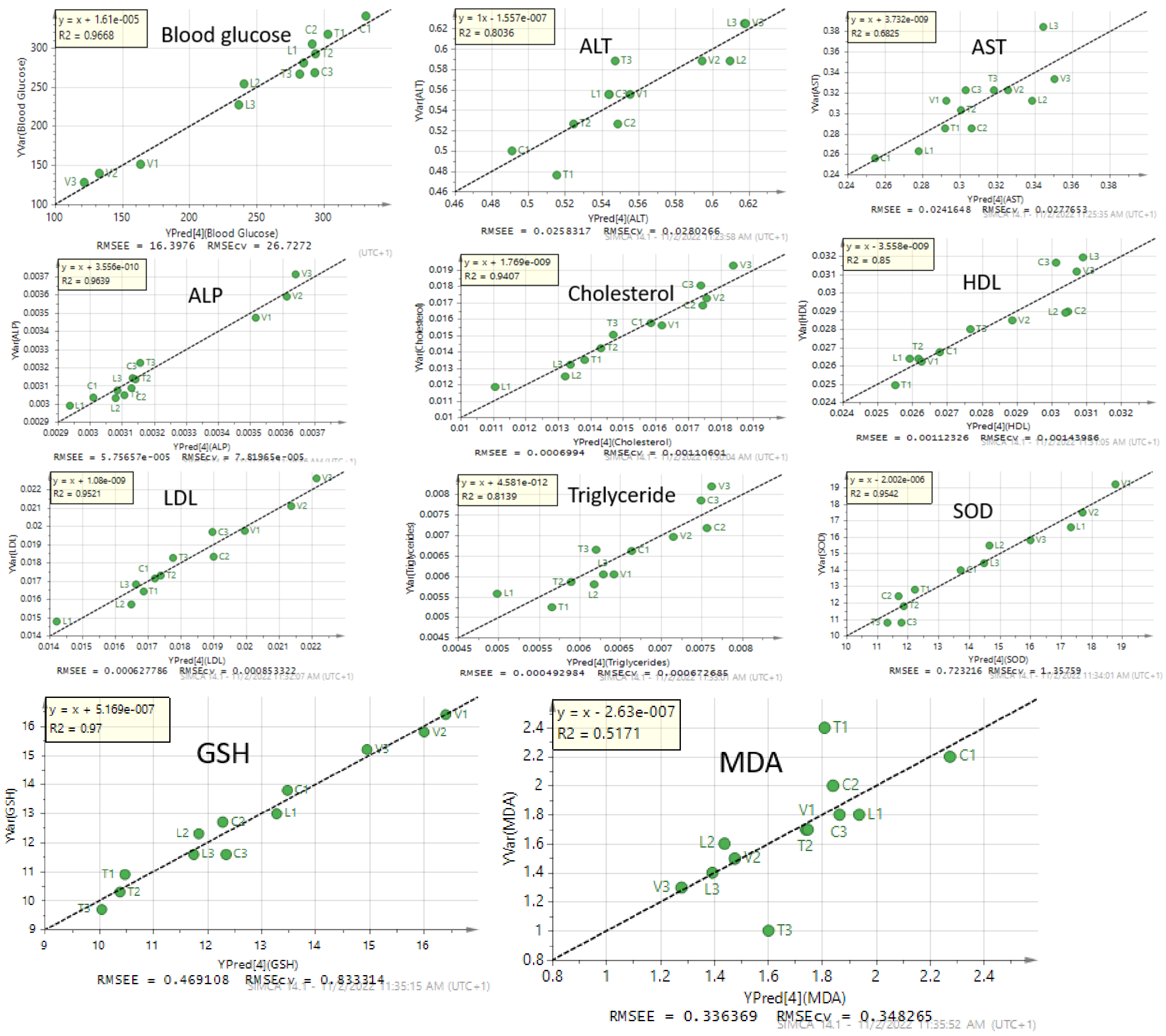

Figure S2. Y-observed versus Y-predicted plot showing the constructed model's goodness of fit based on metabolites quantified by NMR in legume sprouts against values of various biomarkers used for evaluation of antidiabetic effect.

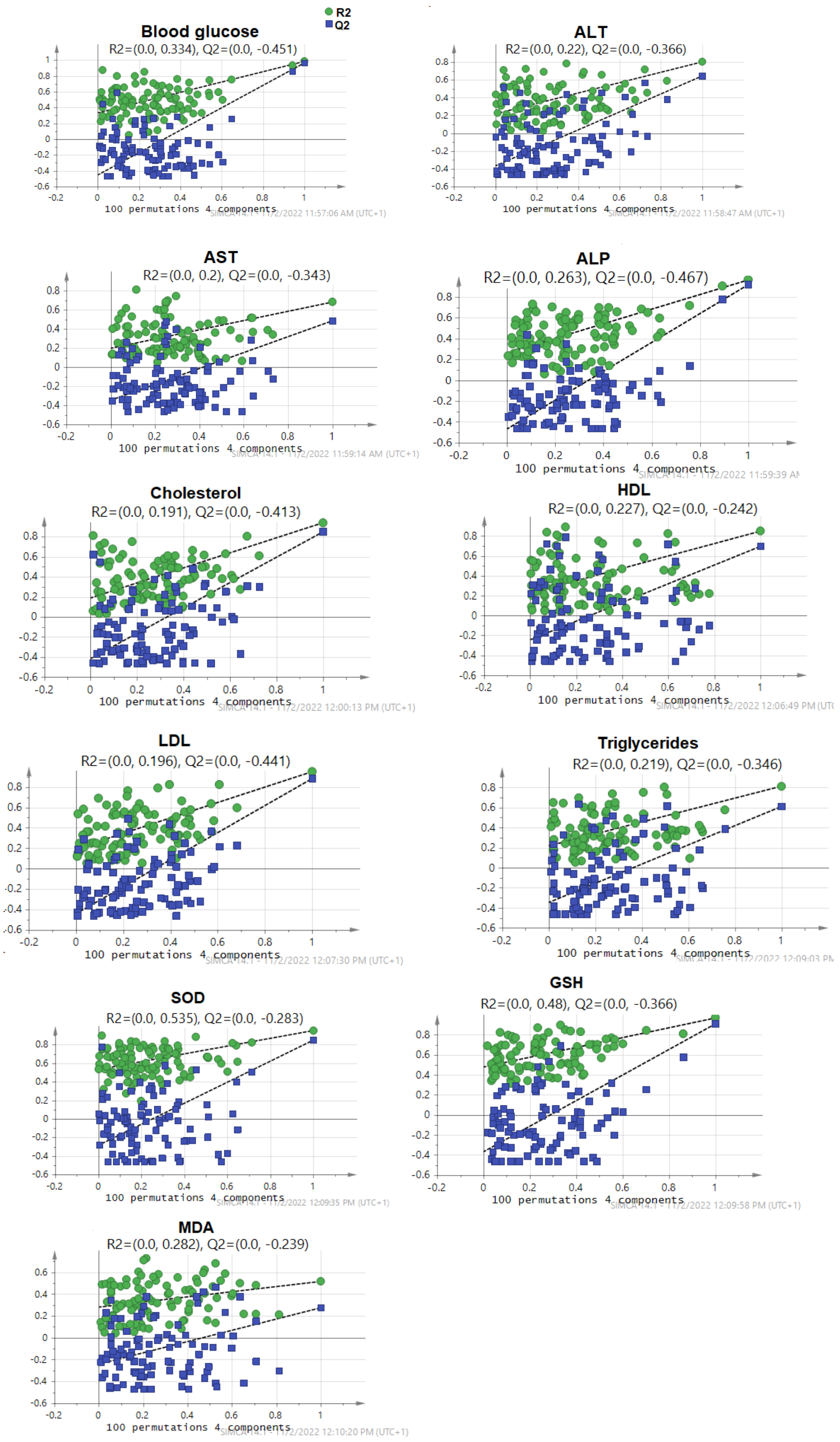

Figure S3: Permutation tests (n=100) of the various antidiabetic biomarkers modelled for phytochemicals identified by NMR in legume sprouts.
